# Metabolomic Profile, Antioxidant Capacity, and Preliminary Cellular Activity of *Krugiodendron ferreum* (Vahl) Urb., a Traditional Plant from Yucatan

**DOI:** 10.3390/molecules31142478

**Published:** 2026-07-15

**Authors:** Brenda Pacheco-Hernández, Teresa Ayora-Talavera, Julia Cano-Sosa, Neith Pacheco, Emanuel Herrera-Pool, Ángel Virgilio Domínguez-May, Lilia G. Noriega, Martha Guevara-Cruz, Isabel Medina-Vera, Azalia Avila-Nava

**Affiliations:** 1 Centro De Investigación y Asistencia en Tecnología y Diseño del Estado de Jalisco, A.C. Subsede Sureste, Parque Científico Tecnológico de Yucatán, Sierra Papacal-Chuburná Puerto, Mérida 97302, Yucatan, Mexico; brpacheco_al@ciatej.edu.mx (B.P.-H.); jcano@ciatej.mx (J.C.-S.); npacheco@ciatej.mx (N.P.); ivherrera_al@ciatej.edu.mx (E.H.-P.); 2 Unidad de Investigación, Hospital Regional de Alta Especialidad de la Península de Yucatán, Servicios de Salud del Instituto Mexicano del Seguro Social para el Bienestar (IMSS-BIENESTAR), Mérida 97130, Yucatan, Mexico; 3 TecNM, Instituto Tecnológico Superior del Sur del Estado de Yucatán (ITSSY), Oxkutzcab 97880, Yucatan, Mexico; adominguez@suryucatan.tecnm.mx; 4 Departamento de Fisiología de la Nutrición, Instituto Nacional de Nutrición y Ciencias Médicas Salvador Zubirán, Ciudad de México 14080, Mexico; lilia.noriegal@incmnsz.mx (L.G.N.); martha.guevarac@incmnsz.mx (M.G.-C.); 5 Departamento Metodología de Investigación, Instituto Nacional de Pediatría, Ciudad de México 04530, Mexico; imedinav@pediatria.gob.mx

**Keywords:** medicinal plant, phenolic acids, antioxidants, hormesis, UPLC-PDA-ESI-MS

## Abstract

*Krugiodendron ferreum* (Vahl) Urb. (KF), commonly known as Ch’iin took’, is a species native to the Yucatan Mexico, whose phytochemical composition remains poorly characterized. Thus, the aim of this study was to evaluate the metabolomic profile, antioxidant capacity, and preliminary cellular activity of KF. An aqueous extract was obtained from KF bark (20 mg/mL). Antioxidant capacity was evaluated using ORAC and DPPH assays. Total phenolic content was evaluated by the Folin–Ciocalteu method. The phenolic profile was determined by UPLC-PDA-MS, and preliminary cellular activity was evaluated by estimating cell number based on the quantification of DAPI-stained nuclei after 72 h of treatment with extract concentrations ranging from 3 to 3000 μg/mL. The extract showed an antioxidant capacity of 1765 ± 59.1 Trolox equivalents/mL and 85.9 ± 0.34% of DPPH radical scavenging activity. The total polyphenol content was 1163 ± 61.5 mg GAE/L, and its phenolic profile was mainly characterized by catechin 3-O-rhamnoside, taxifolin 3-O-rhamnoside, and myricetin-3-O-rhamnoside. Cell activity analysis revealed that both the 3 and 300 μg/mL concentrations increased the number of cells and the area covered by them compared with the control group at 48 and 72 h. The 3000 μg/mL concentration showed no difference compared with the water control. Our results suggest that KF contains polyphenolic compounds and antioxidant properties without affecting cellular response.

## 1. Introduction

Plants are fundamental for the development of modern medicine and play an important role in drug discovery based on the presence of phytochemicals and their contribution to drug discovery [1]. In recent decades, ethnomedical research has become an essential approach for identifying plant species with therapeutic potential, providing a scientific basis for the discovery of metabolites with biological activity. Among these, polyphenols have attracted particular attention because of their antioxidant properties and their role in the prevention and management of chronic diseases [2]. However, many plant species with ecological and cultural relevance remain chemically underexplored, limiting the understanding of their metabolite diversity and biological potential [3,4].

Mexico is a country with great biodiversity, with more than 23,000 reported plant species, approximately 3000 of which are related to medicinal properties [5]. Yucatan, Mexico represents an important reservoir of this biodiversity by the presence of native and endemic plants that have been traditionally used by Mayan communities for generations [6,7,8]. Thus, the study of these species provides an opportunity to characterize their phytochemical composition and validate their potential biological activities.

*Krugiodendron ferreum* (Vahl) Urb. (KF) is a species native to this region, belonging to the Rhamnaceae family. It is locally known as Ch’iin took’ in the Mayan language and is valued for the exceptional density of its wood and traditional applications in Mayan communities [2,9,10,11,12]. Despite cultural relevance, there is little scientific evidence about the effects of KF. Previous studies have demonstrated that aqueous bark extracts exhibit bactericidal and antibiofilm activity against resistant *Staphylococcus aureus* strains [13,14,15]. More recent studies have also been showed the ant virulence and resistance-modifying properties, including inhibition of biofilm formation, hemolysis, and potential interactions with the NorA efflux pump [16].

Species of the Rhamnaceae family are known to contain a wide range of secondary metabolites, including flavonoids, triterpenes, alkaloids, tannins, and other phenolic compounds that have been associated with antioxidant and other pharmacological activities [17,18]. In KF, preliminary phytochemical screening has identified alkaloids, tannins, terpenes, and saponins, while gas chromatography–mass spectrometry (GC–MS) analyses have detected compounds such as neophytadiene, phytol, phytol acetate, squalene, lupeol, lupeol trimethylsilyl ether, α-tocospiro B, α-tocospiro (TMS derivative), vitamin E (α-tocopherol), and D-α-tocopherol succinate [15,16]. However, despite these initial findings, KF remains incompletely characterized. Therefore, the aim of this study was to characterize the metabolomic profile of the aqueous bark extract of KF by Ultra-Performance Liquid Chromatography (UPLC) coupled with Photodiode Array Detection (PDA) and Electrospray Ionization Mass Spectrometry (ESI-MS) methods (UPLC-PDA-ESI-MS), evaluate its antioxidant capacity, and investigate its preliminary cellular activity through nuclear quantification using DAPI staining.

## 2. Results and Discussion

### 2.1. Physicochemical Characteristics of the Aqueous Extract of K. ferreum

The visual appearance and colorimetric parameters of the AE of KF are presented in Table 1. The extract exhibited an orange/brown coloration, which is consistent with the presence of phenolic compounds commonly associated with woody tissues, particularly tannins and lignans. This could be associated with the decoction method, which facilitated the release of water-soluble phenolic compounds from the lignified matrix of the bark. In addition, the color characteristics may also be related to its antioxidant properties. Similar observations have been reported in previous studies, in which high temperatures increased the extraction of phenolic compounds [19]. In fact, decoction has been described as one of the most widely used conventional methods and is considered an effective method for extracting components from hard and fibrous parts of plants, such as the bark or roots [20,21].

The extract had a pH of 6.45, which could promote the stabilization of polyphenols [22]. Tannins, which are characteristic compounds of many barks, are more stable in acid environment than in neutral or basic (pH 7–10) [23,24].

### 2.2. Antioxidant Capacity and Total Phenolic Content of the Aqueous Extract of K. ferreum

The antioxidant capacity of the AE of KF was determined by the ORAC and DPPH assays (Table 2). In the ORAC assay, the antioxidant activity of AE of KF was similar to other traditional plant extracts, such as those of *Laurus nobilis* L. (1896.10 ± 8.77 µmol TE/mL), *Thymus serpyllum* L. (1734.74 ± 8.77 µmol TE/mL) [25,26].

In the DPPH assay, the AE of KF scavenged nearly 86% of this radical (Table 2). These findings are higher than those reported for bark extracts from *Ficus carica*, whose scavenging activity ranged from 48.98 ± 0.42 to 48.55 ± 0.84% [27], as well as different concentrations of *Ilex dipyrena* bark, whose inhibition percentages ranged between 51.00 ± 0.00% and 69.00 ± 1.16% [28]. However, compared with *Prunus africana* bark, KF also had higher DPPH radical scavenging activity, which was 76.76 ± 8.62% [29], with KF slightly above this value.

The antioxidant activity observed in KF may be related to polyphenolic compounds, which are the main contributors to this activity but not the only compounds involved, as other phytochemicals, such as carotenoids, terpenoids, alkaloids, and vitamin-like compounds, among others, may also contribute [30,31]. The total phenolic content of the AE of KF (Table 2) was similar to that reported for other traditional plant barks, such as *Prunus africana* bark (189.0 ± 16.93 mg GAE/g) and *Betula pendula* bark (440.74 mg GAE/g) [29,32]. These findings are consistent with those of previous reports indicating that the bark of woody plants contains a high proportion of polyphenols, including tannins and lignin [32,33]. Furthermore, it has been reported that extraction using polar solvents, such as water, which is widely employed in traditional practices, facilitates the recovery of hydrophilic compounds, including flavonoids and tannins [34], which are metabolites that are often present at high concentrations in bark. Indeed, evidence suggests that bark tannins are readily extracted using polar solvents, such as water or water–ethanol mixtures, which may explain the high total phenolic content observed in the AE of KF [35]. Furthermore, considering the traditional drying method used in this study, it is possible that the decrease in enzymatic degradation and oxidative processes favored the preservation and preparation of phenolic constituents and other secondary metabolites in the bark [36].

In recent years, the important role of woody plants has been highlighted [21]. Bark contains significant amounts of secondary metabolites with strong antioxidant properties, as demonstrated in different studies [32]. In this regard, findings regarding the bark of the KF plant revealed the presence of bioactive compounds with antioxidant activity.

It is widely recognized that antioxidant capacity and polyphenols play important roles in oxidative stress and inflammation in various metabolic disorders, such as type 2 diabetes (T2D) [37,38], nonalcoholic fatty liver disease [39], neurodegenerative diseases [40], hypertension [41], metabolic syndrome [42], chronic kidney disease [43], and urolithiasis [44]. However, in the case of KF, further studies are needed to determine the biological activities of the plant and its phytochemical constituents.

### 2.3. Phenolic Profile of the Aqueous Extract of the Bark of K. ferreum by UPLC-PDA-ESI-MS

**Figure 1:**
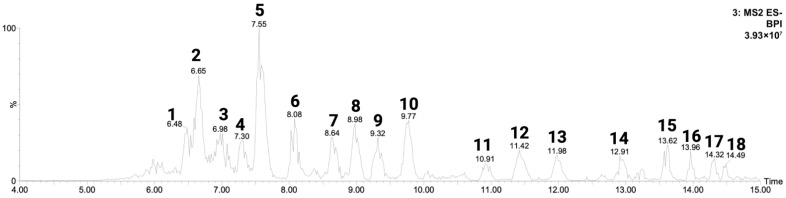
Chromatogram of phenolic profile identified in aqueous extract of *K. ferreum* bark obtained by UPLC-ESI-MS analysis.

The phenolic profile of the AE of KF revealed 18 resolved signals by mass spectrometric detection (Figure 1). However, the UPLC–PDA trace (Supplementary Figure S1) displayed a broad unresolved chromatographic envelope, indicating that several compounds contributed to overlapping UV responses due to coelution. The tentative identification of the compounds (MSI level 2) was carried out primarily on the basis of mass spectrometry data, considering precursor ions, MS fragmentation patterns and comparisons with reports previously described in the specialist literature (Figure 1).

**Figure 2:**
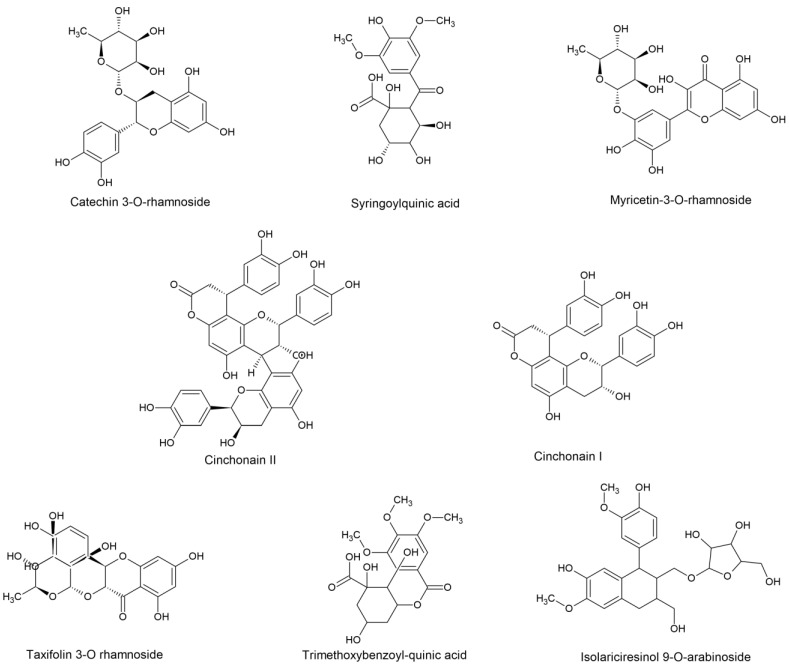
Representative structures of selected tentatively identified compounds (MSI level 2) from the aqueous extract of *K. ferreum*.

Among the 18 signals detected, we identified 15 tentative compounds (MSI level 2) (Table 3), which are represented in Figure 2. Detailed MS fragmentation spectra for all tentatively identified compounds are presented in Supplementary Figure S2.

Within the retention time (RT) range of 8.98–12.91 min, multiple compounds with a molecular ion at *m*/*z* 449 ([M − H]^−^) were detected and tentatively identified as isomers of taxifolin 3-O-rhamnoside. Their spectra showed fragments at *m*/*z* 303, 297, 285, 270, 259, 257, 242, 235, 226, 220, 219, 199, 179, 165, 161, 152, 151, 149, 137, and 125. The *m*/*z* 303 fragment corresponds to taxifolin in the aglycone form following the loss of a rhamnose unit (146 Da) [45]. The differences observed between the spectra suggest that different isomeric forms are formed by different fragmentation pathways.

Compounds with a molecular ion at *m*/*z* 463 ([M − H]^−^) were also detected in the RT range of 13.24–14.49 min and were tentatively identified as isomers of myricetin 3-O-rhamnoside. The main fragments observed were *m*/*z* 317, 299, 289, 284, 272, 180, 179, 152, and 151. The *m*/*z* 317 fragment corresponds to myricetin in the aglycone form following the loss of a rhamnose unit (146 Da) [46,47]. Taxifolin 3-O-rhamnoside and myricetin-3-O-rhamnoside were the compounds that repeatedly predominated at different retention times. Taxifolin 3-O-rhamnoside is a flavanonol that has demonstrated beneficial effects on metabolic parameters in animal models of T2D [48]. Myricetin-3-O-rhamnoside is a glycosylated derivative of myricetin. Studies specifically focused on this compound remain limited. Myricetin-3-O-rhamnoside exhibited high antioxidant activity in the DPPH assay, with an IC_50_ of 1.4 μg/mL, which was higher than that of vitamin E (IC_50_ 3 μg/mL), which was used as a positive control. Furthermore, this compound modulated the expression of genes associated with the oxidative stress response, DNA damage repair and the regulation of apoptosis, suggesting a potential multifunctional cytoprotective effect at the cellular level [49]. In contrast, preclinical studies have shown that myricetin has antimicrobial, anti-infective, antioxidant, and anti-inflammatory effects [50,51]. However, these activities should be interpreted as reported for the pure compound and not as direct evidence for the biological activity of the extract.

At an RT of 7.55 min, a molecular ion at *m*/*z* 435 ([M − H]^−^) was detected and tentatively assigned as catechin 3-O-rhamnoside. Fragments were observed at *m*/*z* 313, 289, 271, 245, 150, 137, and 125. The *m*/*z* 289 fragment corresponds to the aglycone form of catechin following the loss of a rhamnose unit (146 Da), whereas the other fragments are associated with secondary fragmentation of the flavan-3-ol skeleton [52,53]. Catechin 3-O-rhamnoside is a glycosylated flavan-3-ol that belongs to the catechin family. While catechins have been extensively studied, rhamnoside derivatives are less well documented in the literature and are more rarely distributed in plant matrices. Its presence has been reported in the bark of *Lannea kerstingii* and *Bruguiera gymnorrhiza,* where it exhibits antimicrobial activity [52,53]. These reports refer to isolated compounds or related species and should not be directly extrapolated to the biological activity of KF extract.

At an RT of 8.64 min, a molecular ion at *m*/*z* 371 ([M − H]^−^) was detected, tentatively identified as syringoylquinic acid, with fragments at *m*/*z* 353, 249, and 231. Syringoylquinic acid is a quinic acid-derived phenolic acid classified as a hydroxybenzoylquinic acid, previously reported in celery leaves (*Apium graveolens* L.) [54]. Another quinic acid derivative was identified at an RT of 9.32 min and exhibited a deprotonated molecular ion at *m*/*z* 385 ([M − H]^−^). The MS spectrum showed characteristic fragment ions at *m*/*z* 267 and 249, which allowed its tentative assignment as trimethoxybenzoylquinic acid, an acylated quinic acid derivative previously reported in *Ziziphus* leaves [55]. Both belong to the group of quinic acid derivatives. These compounds have a structure consisting of a quinic acid core esterified with methoxylated aromatic acids, specifically syringic acid in the case of syringoylquinic acid and a trimethoxybenzoic acid derivative for trimethoxybenzoylquinic acid. Syringoylquinic acid contains a syringyl group derived from syringic acid, a compound to which antioxidant, anti-inflammatory, hepatoprotective, and neuroprotective properties have been attributed [56]. However, specific biological evidence for both metabolites is limited and derived primarily from structurally related compounds.

At an RT of 6.48 min, a molecular ion at *m*/*z* 739 ([M − H]^−^) was detected, tentatively identified as cinchonain II. The compound exhibited fragments at *m*/*z* 723, 721, 613, 587, 569, 451, 413, 313, 299, 287, 273, 255, 245, 243, 177, 161, 137, and 125. This pattern is consistent with previous reports for cinchonain-type structures, although the exact fragmentation mechanism requires further study. It was not possible to determine the type of isomerism [57]. Similarly, at an RT of 6.65 min, a molecular ion at *m*/*z* 451 ([M − H]^−^) was observed, tentatively identified as cinchonain I, with fragments at *m*/*z* 313, 305, 287, 161, 137 and 125. This finding is similar to that observed for cinchonain II [58]. Cinchonains are a group of flavanolignans that have been reported in several plant species, including *Smilax corbularia*, *Trichilia catigua*, *Uncaria tomentosa*, and *Apocynum cannabinum*, as well as in different plant parts, such as leaves, roots, fruits, stem bark, rhizomes, aerial parts, twigs, wood, and flowers [59]. These compounds have previously been associated with anti-inflammatory effects [59]. According to Fahsi et al., cinchonains have been identified in approximately 60 plant species belonging to 27 botanical families, including Rosaceae, Meliaceae, Smilacaceae, Rubiaceae, Ericaceae, Moraceae, Apocynaceae, Liliaceae, Rhizophoraceae, Polygonaceae, Bignoniaceae, Fabaceae, Sapindaceae, Hypericaceae, Urticaceae, Bombacaceae, Theaceae, Asteraceae, Berberidaceae, Asclepiadaceae, Myristicaceae, Euphorbiaceae, Lauraceae, Fagaceae, Tiliaceae, Erythroxylaceae, and Podocarpaceae. Therefore, the available information suggests that cinchonains have rarely been reported in species belonging to the Rhamnaceae family [59]. Furthermore, a recent study demonstrated that cinchonaine significantly reduced the expression of inflammatory molecules and reactive oxygen species, suggesting possible anti-inflammatory and antioxidant effects [60]. However, these effects were observed in isolated experimental systems, and their relevance within complex plant extracts requires further investigation.

**Table 3:** Tentatively identified phenolic compounds (MSI level 2) were identified in the aqueous extract of *K. ferreum*.

No.	RT	[M – H]^−^	Molecular Formula	Fragments (*m*/*z*)	Tentative Identification of Compounds (MSI Level 2)	Ref.
1	6.48	739	C_39_H_32_O_16_	723, 721, 613, 587, 569, 451, 413, 313, 299, 287, 273, 255, 245, 243, 177, 161, 137, 125	Cinchonain II	[58]
2	6.65	451	C_24_H_20_O_9_	313, 305, 287, 161, 137, 125	Cinchonain I	[57]
3	6.98	723		601, 593, 583, 571, 553, 465, 435, 305, 289, 287, 273, 245, 161, 137, 125	Unknown	
4	7:30	769		723, 651, 593, 401, 305	Unknown	
5	7.55	435	C_21_H_24_O_11_	313, 289, 271, 245, 150, 137, 125	Catechin 3-O-rhamnoside	[52,53]
6	8.08	351		289.249	Unknown	
7	8.64	371	C_16_H_19_O11_11_	353, 249, 231	Syringoylquinic acid	[55]
8	8.98	449	C_21_H_22_O_11_	285, 259, 235, 220, 219, 165, 149, 137, 125	Taxifolin 3-O rhamnoside (isomer 1)	[45]
9	9.32	385	C_17_H_21_O_11_	267, 249	Trimethoxybenzoyl-quinic acid	[55]
10	9.77	449	C_21_H_22_O_11_	303, 270, 257, 242, 226, 199, 161, 137, 125	Taxifolin 3-O rhamnoside (isomer 2)	[45]
11	10.91	449	C_21_H_22_O_11_	303, 297, 179, 152, 151, 125	Taxifolin 3-O rhamnoside (isomer 3)	[45]
12	11.42	449	C_21_H_22_O_11_	303, 285, 179, 151, 125	Taxifolin 3-O rhamnoside (isomer 4)	[45]
13	11.98	491	C_25_H_32_O_10_	359, 341,327	Isolariciresinol 9′-O-alpha-L-arabinofuranoside	[61]
14	12.91	449	C_21_H_22_O_11_	303, 297, 259, 179, 125	Taxifolin 3-O rhamnoside (isomer 5)	[45]
15	13.62	463	C_21_H_20_O_12_	317, 299, 289, 180, 179, 152, 151	Myricetin-3-O-rhamnoside (isomer 1)	[46,47]
16	13.96	463	C_21_H_20_O_12_	317, 299, 284, 272, 179	Myricetin-3-O-rhamnoside (isomer 2)	[46,47]
17	14.32	463	C_21_H_20_O_12_	317, 299, 289, 180, 179, 152, 151	Myricetin-3-O-rhamnoside (isomer 3)	[46,47]
18	14.49	463	C_21_H_20_O_12_	317, 299, 289	Myricetin-3-O-rhamnoside (isomer 4)	[46,47]

RT: Retention time.

At an RT of 11.98 min, a molecular ion at *m*/*z* 491 ([M − H]^−^) was observed, tentatively identified as isolariciresinol 9-O-arabinoside. The main fragments detected were *m*/*z* 359, 341 and 327. The *m*/*z* 359 fragment corresponds to the loss of a rhamnosyl group [61]. Hydroxybenzoic acids have been described for their antioxidant, anti-inflammatory and antimicrobial effects [62]. Isolariciresinol 9′-O-alpha-L-arabinofuranoside is a glycosylated lignan characteristic of the woody bark of Atacama and Blesbok sweet potato (*Ipomoea batatas* L.) [61]. Lignans possess antioxidant, antibacterial, and antiviral properties [63]. Furthermore, studies have highlighted the importance of the interaction between lignans and the gut microbiota and their biotransformation into enterolignans; these interactions may be primarily responsible for the biological activities associated with these compounds [63]. In addition, flavonoids, tannins, and polyphenolic lignans have been shown to have medicinal effects on urolithiasis and renal cell damage [44,64]. However, these effects have been demonstrated for isolated compounds or structural analogs, and further studies are needed to determine their contributions to complex plant extracts.

Notably, this is the first study to evaluate the polyphenol profile, as Dzul et al. identified compounds using GC–MS and identified volatile, lipid, and derivatized compounds, including fatty acids, terpenes, sterols, tocopherols, sugars, and derivatized polyols [16].

### 2.4. Effects of the Aqueous Extract of K. ferreum on Cell Activity

**Figure 3:**
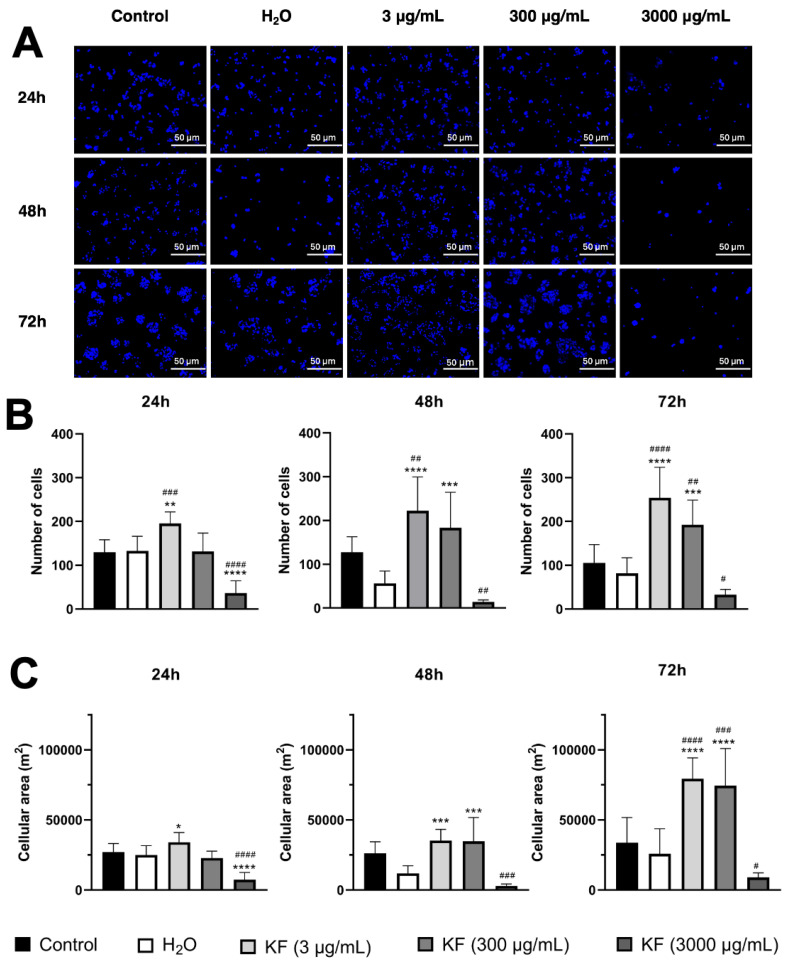
Effect of aqueous extract of *K. ferreum* on NCI-N87 cell line parameters at 24 h, 48 h, and 72 h. (**A**) Representative fluorescence images of nuclei stained with DAPI; (**B**) Cell number by DAPI-stained nuclei counting and (**C**) Cellular area quantified from DAPI-stained fluorescence images. Data are presented as the mean and standard deviation. Statistical analysis was by one-way ANOVA following by Dunn’s test. Statistical significance was set at * *p* < 0.05; ** *p* < 0.01; *** *p* < 0.001; **** *p* < 0.0001 and # *p* < 0.05; ## *p* < 0.01; ### *p* < 0.001; #### *p* < 0.0001. Comparison with the medium control (#) and the water control (*). KF: *K. ferreum*. Experiment was evaluated by duplicate in two independent experiments.

The results revealed that cellular confluence decreased as the concentration of the AE of KF increased. The results were similar when different extract concentrations were compared at 24, 48, and 72 h (Figure 3A). Cellular activity was assessed through the quantification of DAPI-stained nuclei, these findings suggest that low concentrations of the extract did not adversely affect cell number and were associated with an increase in the number of detected nuclei. In fact, recent studies on the AE of this plant’s bark have shown that a concentration of >1000 μg/mL is required to reduce cell viability by 50% within 48 h in Vero CCL-81 cells [16]. Other studies on the Rhamnaceae family have reported mixed results; a study of the effect of the AE of *Zizyphus joazeiro* bark on Vero cell viability using MTT assays revealed that cell viability decreased at concentrations of 0.55 mg/mL and above, with an IC_50_ of 1.4 mg/mL [65]. Moreover, a study of *Ampelozizyphus amazonicus* Ducke bark extract, evaluated in A20 lymphoma cell cultures at 48 and 72 h, revealed no differences compared with the negative control at concentrations ranging from 150 to 0.01 μg/mL [66].

The quantification of DAPI-stained nuclei revealed that after 24 h of treatment, compared with both the culture medium control and the water control, a concentration of 3 μg/mL resulted in a greater number of cells and greater cell-covered area (*p* < 0.05) (Figure 3B,C). Moreover, compared with the controls, the concentration of 3000 μg/mL resulted in a lower number of cells and a smaller cell-covered area (*p* < 0.0001) (Figure 3B,C). At 48 and 72 h, compared with the water control, the treatments with 3 μg/mL and 300 μg/mL maintained higher cell numbers and cell-covered areas (Figure 3B,C). Although the concentration of 3000 μg/mL resulted in lower cell numbers and cell-covered areas than the culture medium control did (*p* < 0.05) at both time points (48 and 72 h), no differences were observed relative to the water control (Figure 3B,C). These observations indicate that low concentrations of the extract were associated with an increased number of detected nuclei, whereas the highest concentration evaluated resulted in a reduced nuclear count. Therefore, the phenomenon we observed may correspond to ‘hormesis or low-dose stimulation’. Thus, low concentrations of the extract significantly increased the cell number, whereas compared with the water control, high concentrations did not affect the cell number or negative effects across the two time points (48 and 72h) evaluated (*p* < 0.05). Murakami A. describes hormesis as a phenomenon of adaptation in which a stressor induces cellular stress responses at low or moderate doses, whereas at high doses, it causes inhibition. Furthermore, evidence of the effects of polyphenols, which exhibit physiological effects via hormesis, is highlighted, with biphasic dose responses from resveratrol, quercetin, and type B procyanidins being beneficial for ameliorating the effects of hemodynamics in the central nervous system, demonstrating that high doses of polyphenols are potentially toxic [67].

This study provides new preliminary in vitro evidence regarding the effects of KF, a plant widely used by the Maya population of Yucatan, Mexico but poorly studied. Our findings support the relevance of further investigations of plants, such as KF. Although several compounds were tentatively annotated (MSI level 2) in the phenolic profile, additional studies are needed to better understand the biological activities of compounds in appropriate experimental models. Future in vivo studies will be important to further evaluate their efficacy, safety, and bioavailability, providing a more comprehensive understanding of their biological relevance.

### 2.5. Limitations and Future Prospects

To the best of our knowledge, this is the first study to characterize the metabolite profile, antioxidant capacity, total phenolic content, and preliminary cellular activity of KF. These findings provide an initial scientific basis for describing the in vitro antioxidant activity and preliminary cellular effects of this species, contributing to its chemical characterization and supporting further research.

However, certain limitations must be considered when interpreting the results. One of these is the use of the Folin–Ciocalteu assay; although this method is widely used to estimate total phenolic content, it is not entirely specific to phenolic compounds, as it is based on the reduction of the reagent by electron-donating substances. Therefore, other reducing compounds present in the extract may contribute to the measured signal, potentially leading to an overestimation of the total phenolic content. Additionally, chemical characterization was performed using UPLC–ESI–MS, which is based on exact mass, MS fragmentation patterns, chromatographic behavior, and comparisons with data published in the literature and specialized databases. The integration of these criteria allowed for the identification of compounds with a high degree of confidence; however, structural confirmation of the identified metabolites would require the analysis of authentic reference standards and complementary techniques.

In addition, a limitation of this study is that the bark samples were collected during a single sampling event. Nevertheless, according to the phenology of the species, sampling was performed in January, when the plants were in the vegetative stage and did not exhibit flowering or fruiting [68]. This sampling strategy likely reduced the variability associated with reproductive physiological changes, although seasonal variation in metabolite composition cannot be excluded. Future studies should evaluate samples collected across different seasons and phenological stages to determine the influence of seasonal and phenological variations on the phytochemical composition and cellular activity of KF bark. Although this information is provided in the plant collection section, it is now explicitly highlighted as a limitation of the study, and the results should therefore be interpreted as preliminary. Cellular evaluation was conducted using the NCI-N87 cell line as a preliminary in vitro model to explore the effects of the AE on cell activity in a human epithelial cancer context. Although this approach provides initial insights into cellular responses, further studies using non-cancerous cell lines are warranted to better characterize the cellular effects of KF extracts in the context of the traditional uses reported for this species.

In perspective, the results obtained provide a foundation for further research aimed at gaining a better understanding of the chemical constituents of KF and evaluating its antioxidant activity in vitro using complementary cellular models. Subsequent studies in animal models will allow for a deeper exploration of aspects related to the efficacy, safety, and bioavailability of these compounds, assessments that are essential for understanding the effects of traditional plants.

## 3. Materials and Methods

### 3.1. Experimental Reagents and Instruments

Reagents used included Folin–Ciocalteu reagent (Sigma-Aldrich, St. Louis, MO, USA), 2,2-diphenyl-1-picrylhydrazyl (DPPH) (Sigma-Aldrich, St. Louis, MO, USA), fluorescein (Sigma-Aldrich, St. Louis, MO, USA), 2,2′-azobis(2-methylpropionamidine) dihydrochloride (AAPH) (Sigma-Aldrich, St. Louis, MO, USA), Trolox (Sigma-Aldrich, St. Louis, MO, USA), gallic acid (Sigma-Aldrich, St. Louis, MO, USA), sodium carbonate (Na_2_CO_3_; J.T.Baker, Avantor Performance Materials, Center Valley, PA, USA). Dulbecco’s modified Eagle’s medium (DMEM) (Gibco, Thermo Fisher Scientific, Grand Island, NY, USA), fetal bovine serum (Gibco, Thermo Fisher Scientific, Grand Island, NY, USA), penicillin–streptomycin (Sigma-Aldrich, St. Louis, MO, USA), amphotericin B solution (Sigma-Aldrich, St. Louis, MO, USA), trypsin–EDTA solution (Sigma-Aldrich, St. Louis, MO, USA)**,** Dulbecco’s phosphate-buffered saline (DPBS) (Sigma-Aldrich, St. Louis, MO, USA)**,** NucBlue™ Fixed Cell Stain ReadyProbes™ Reagent (DAPI; Invitrogen Carlsbad, CA, USA), and the NCI-N87 human gastric cancer cell line (Científica SENNA, Mexico City, Mexico) were used. All the reagents were of analytical grade. Purified water was used throughout the experiments.

Instrumentation: Color measurements were performed using a Konica Minolta CM-5 spectrophotometer (Konica Minolta Inc., Tokyo, Japan). The pH determination was carried out using a Thermo Scientific™ Orion Star™ A111 potentiometer (Thermo Fisher Scientific, Waltham, MA, USA). The absorbance and fluorescence were measured using a BioTek Synergy HT microplate reader/spectrofluorometer (BioTek Instruments, Winooski, VT, USA). A CO_2_ incubator (New Brunswick™ Galaxy^®^ 48 R, Eppendorf, Hamburg, Germany) was used. A Waters Acquity H-Class UPLC system equipped with a quaternary pump, automatic injector, and PDA λ detector (Waters, Milford, MA, USA) was used. An Acquity CORTECS UPLC BEH C18 column (1.6 μm, 100 × 3.0 mm ID) (Waters, Milford, MA, USA) was used. Xevo TQ-S micro mass spectrometer (Waters, Milford, MA, USA) was used.

### 3.2. Plant Collection and Identification of K. ferreum

**Figure 4:**
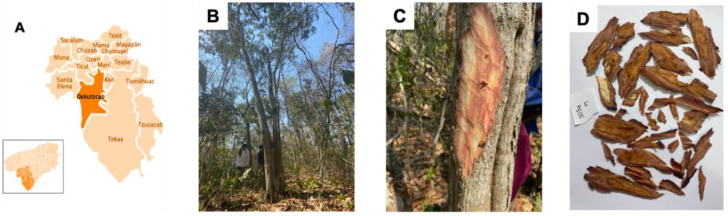
Collection of the medicinal plant sample. (**A**) Location of the plant collection. (**B**) Complete KF plant. (**C**) Obtaining the bark from the plant. (**D**) Weighing and drying the bark of the KF plant.

The plant material used for the extraction was the bark of the KF. The KF bark was collected in Xohuayan, Oxkutzcab, Yucatán (20°11′16.4″ N 89°23′13.6″ W) (Figure 4A) on 10 January 2025, with permission granted by the informant and owner of the cornfield. The tree was approximately 23.87 cm in diameter and approximately 5 m in height (Figure 4B). The habitat conditions during collection were a temperature of 31 °C, a humidity of 59%, an atmospheric pressure of 760 mm Hg, and an altitude of 103 m above sea level. The tree trunk with the bark partially removed, revealing a reddish color on the exposed surface (Figure 4C). It was then dried at room temperature (Figure 4D). For botanical identification and registration of the plant, a 28 cm × 40 cm branch with leaves was used. The plant was subsequently identified and confirmed as *K. ferreum* (Vahl) Urb. by a certified biologist. The specimen was added to the CICY herbarium collection with accession number 078778 and registered in the CONABIO (National Commission for the Knowledge and Use of Biodiversity) database.

### 3.3. Preparation of Aqueous Extract of K. ferreum

The bark of KF was dried at room temperature, without direct exposure to sunlight, under conditions of relative humidity between 52 ± 3% and a temperature between 23 and 27 °C. The drying process was monitored until a constant weight was reached, and a total water loss of 27.6% was recorded.

The extract was prepared from the dried bark of the KF plant using decoction as the extraction method to obtain an AE. One gram of dried bark was added to 50 mL of purified water (at a concentration of 20 mg/mL), following the informant’s instructions for consumption, and it was boiled at a temperature of 97 ± 3 °C for approximately 35 min. It was then filtered to remove plant material residues, without pH adjustment and following conventional preparation practices. In addition, concentrations of 3000 μg/mL, 300 μg/mL, and 3 μg/mL were prepared for the cellular effect studies and related evaluations.

### 3.4. Analysis of Color

The color of the extract was determined by the degree of light transmittance in a Konica Minolta CM-5 spectrophotometer (Konica Minolta, Inc., Tokyo, Japan). For interpretation, L* indicates lightness, and a* and b* represent chromaticity. Negative values of a* indicate a green color, whereas positive values indicate a red color. For b*, negative values represent blue, and positive values represent yellow. From this, the color index is calculated using the formula CI = (1000 × a*)/(L × b*) [69].

### 3.5. Determination of Antioxidant Activity

Antioxidant activity was evaluated using 2,2-diphenyl-1-picrylhydrazyl (DPPH) and oxygen radical absorbance capacity (ORAC) assays.

#### 3.5.1. Radical Scavenging Activity by the 2,2-Diphenyl-1-picrylhydrazyl Radical

To evaluate the antioxidant activity by the 2,2-diphenyl-1-picrylhydrazyl (DPPH) assay, the following mixture was prepared: 50 μL of sample or blank and 2.95 mL of DPPH (1 mM) [70]. The mixtures were incubated in the dark for 20 min at room temperature, after which the absorbance was measured at a wavelength of 517 nm (BioTek, Winooski, VA, USA). The percentage of radical scavenging (%) was calculated as follows:

(1) $$DPPH\;radical\;scavenging\;(\%)\;=\;\frac{Control\;(blank)\;-\;Sample\;(extract)}{Control\;(blank)}\;\times \;100$$

#### 3.5.2. Oxygen Radical Absorbance Capacity

Total antioxidant capacity was measured using the fluorometric oxygen radical absorbance capacity (ORAC) [71]. For this evaluation, the following reaction mixture was placed in a 96-well black plate: 25 μL of extract or standards, 150 μL of fluorescein (40 nM) and 25 μL of 2,2′-azobis(2-methylpropionamidine) dihydrochloride (AAPH) (150 mM). The kinetics were performed for 90 min at an excitation wavelength of 485 nm and an emission wavelength of 520 nm in a Biotek Sinergy HT spectrofluorometer (Biotek, Winooski, VA, USA). Trolox curves were used as standards (0, 8, 20, 40, 60, and 80 μM). The tests were performed in triplicate. The results are expressed as equivalents of Trolox (ET) μmoles ET/mL.

#### 3.5.3. Determination Total Phenolic Content

The total phenol content was measured by the Folin–Ciocalteu spectrophotometric method. Briefly, 50 μL of extract or standard and 250 μL of Folin reagent (2:1) were mixed and then incubated for 5 min in the dark at room temperature. Next, 500 μL of Na_2_CO_3_ (20%) was added, mixed, and incubated for 20 min in the dark at room temperature. Finally, the absorbance at 760 nm was measured using a Biotek Sinergy HT spectrofluorometer (Biotek, Winooski, VA, USA). A gallic acid curve was used as a standard (0–100 mg/L). The tests were performed in triplicate.

### 3.6. Phenolic Profile and Tentative Identification of Compounds by UPLC-PDA-ESI-MS

The chromatographic profiles were obtained using a Waters Acquity H-Class UPLC (Milford, MA, USA) equipped with a quaternary pump (UPQSM), an automatic injector (UPPDALTC), and a PDA λ photodiode array detector (UPPDALTC). Chromatographic separation was performed on an Acquity CORTECS UPLC BEH C18 column (1.6 μm, 100 × 3.0 mm ID) (Waters, Milford, MA, USA) using a mobile phase of 0.1% formic acid in ultrapure water (A) and 0.1% formic acid in acetonitrile (B) under the conditions reported by Herrera-Pool et al. 2021 [72]. The gradient used was as follows: 0–1.5 min, 98.0% A; 2.5 min, 98.0–85.0% A; 6 min, 85–80% A; 1 min, 80–75% A; 3 min, 75.0–50.0% A; 8.0 min, 50.0–5.0% A; 2 min, 5.0% A; 0.5 min, 5.0–75.0% A; 0.5 min, 75.0–85.0% A; 1.0 min, 85.0–98.0% A; and 4 min, 98.0–98.0% A. The flow rate and injection volume were 0.3 mL min^−1^ and 2 µL, respectively. PDA λ readings were taken in the range of 190–400 nm. The absorbance of the analytical response was measured at 290 nm.

Tentative compound annotation was performed using UPLC–DAD–ESI–MS by combining chromatographic RT, UV–visible spectra, accurate mass measurements, precursor ions, and MS fragmentation patterns. Although partial chromatographic co-elution was observed for some constituents, metabolite annotation relied primarily on accurate mass measurements and diagnostic MS fragmentation patterns rather than exclusively on chromatographic peak resolution. The compound assignments were established by comparing the chromatographic behavior, accurate mass, precursor ions, characteristic fragment ions, and MS fragmentation patterns with those previously reported in the literature and available public spectral databases, including MassBank (https://massbank.eu/MassBank/search, accessed on 12 July 2026) and PubChem (https://pubchem.ncbi.nlm.nih.gov/, accessed on 12 July 2026). Isomeric compounds were differentiated on the basis of their retention times and characteristic MS fragmentation profiles. Compound identities were confirmed by comparison with authentic reference standards when available. Otherwise, compounds were classified as putatively annotated compounds (MSI level 2) and therefore considered tentatively identified compounds according to the recommendations of the Metabolomics Standards Initiative [73]. Detailed MS spectra and fragmentation analyses supporting the annotation of all putatively annotated (tentatively identified) compounds are provided in the Supplementary Material (Supplementary Figure S2).

### 3.7. Assessment of Cell Activity by Quantification of DAPI-Stained Nuclei

Cell activity was determined by quantifying 4′,6-diamidino-2-phenylindole (DAPI)-stained nuclei. The N87 cell line obtained from Científica SENNA (Científica SENNA, Mexico City, Mexico) was seeded in 96-well plates at a density of approximately 3.3 × 10^4^ cells per well. A final volume of 200 µL was added to each well, consisting of 180 µL of Dulbecco’s modified Eagle’s medium supplemented with 10% fetal bovine serum, 1% penicillin/streptomycin (100 IU/mL penicillin, 100 µg/mL streptomycin), and 1% amphotericin, together with 20 µL of either control solution (culture medium or water) or AE of KF at final concentrations of 3000, 300, and 3 µg/mL. Each condition was evaluated in duplicate in two independent experiments.

The plates were incubated at 37 °C in a humidified atmosphere containing 5% carbon dioxide (CO_2_). To evaluate the effect of the extract on the cells, the plates were removed from the CO_2_ incubator and analyzed at 24, 48, and 72 h. At each time point, the culture medium was removed, and the cells were washed twice with PBS. The cells were then fixed with 200 µL of 4% paraformaldehyde for 5 min at room temperature. After fixation, the cells were washed twice with PBS and incubated with 25 µL of DAPI solution (1 µg/mL in PBS) for 20 min in the dark.

After staining, the excess dye was removed by washing with PBS, and the nuclei were visualized by fluorescence microscopy using a 20× objective. For each well, at least four randomly selected fields were acquired. The number of DAPI-stained nuclei was quantified using ImageJ software (version 1.54g; NIH, Bethesda, MD, USA). Cell activity was subsequently estimated from the relative number of nuclei present in treated cultures compared with control conditions.

### 3.8. Statistical Analysis

The data were evaluated using the Shapiro–Wilk test to assess normality. The values are expressed as the means ± standard deviations. For quantitative variables, one-way ANOVA followed by Dunn’s test was used to evaluate differences between groups. A two-tailed *p* value < 0.05 was considered to indicate statistical significance. The data were analyzed using GraphPad Prism version 9.3.0 (GraphPad Software, San Diego, CA, USA).

## 4. Conclusions

Our results revealed that the AE of KF exhibited an antioxidant capacity of 1765 ± 59.1 µmol TE/mL and 85.9 ± 0.34% DPPH radical scavenging activity. The total phenolic content was 1163 ± 61.5 mg GAE/L, and the phenolic profile was characterized mainly by catechin 3-O-rhamnoside, taxifolin 3-O-rhamnoside, and myricetin-3-O-rhamnoside. Additionally, cell activity, as determined by DAPI-stained nuclear quantification, increased mainly at concentrations of 3 and 300 μg/mL at 48 and 72 h, respectively, and the concentration of 3000 μg/mL was not lower than that of the water control.

## Figures and Tables

**Table 1:** Qualitative parameters of the aqueous extract of *K. ferreum*.

L*	a*	b*	Color Index	Visual Representation
68.5 ± 0.01	30.9 ± 0.02	48.4 ± 0.01	9.3 ± 0.01	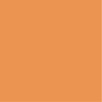

The data are presented as means ± standard deviation. All tests were conducted in triplicate. L*: lightness; a* and b* represent chromaticity.

**Table 2:** Antioxidant activity and polyphenolic content in aqueous extract of *K. ferreum*.

Total Antioxidant Capacity (µmol TE/mL)	Radical Scavenging Activity of DPPH (%)	Total Polyphenols (mg GAE/L)
1765 ± 59.1	85.9 ± 0.34	1163 ± 61.5

The data are presented as mean ± standard deviation. All tests were conducted in triplicate. TE: Trolox equivalents; DPPH: 2,2-diphenyl-1-picrylhydrazyl; GAE: Gallic acid equivalents.

## Data Availability

The original contributions presented in this study are included in the article/Supplementary Material. Further inquiries can be directed to the corresponding authors.

## Supplementary Materials

The following supporting information can be downloaded at: https://www.mdpi.com/article/10.3390/molecules31142478/s1, Supplementary Figure S1. UPLC-PDA chromatographic profile of the AE of *K. ferreum.* Supplementary Figure S2. Tentatively annotated phenolic compounds.

## Abbreviations

The following abbreviations are used in this manuscript:

AAPH	2,2′-Azobis(2-methylpropionamidine) dihydrochloride
ANOVA	Analysis of Variance
CI	Color index
CIELAB	Commission Internationale de l’Éclairage Lab* color space
CONABIO	National Commission for the Knowledge and Use of Biodiversity
CO2	Carbon dioxide
AE	Aqueous extract
KF	*Krugiodendron ferreum* (Vahl) Urb.
DAPI	4′,6-Diamidino-2-phenylindole
DMEM	Dulbecco’s Modified Eagle’s Medium
DPPH	2,2-Diphenyl-1-picrylhydrazyl
ESI	Electrospray Ionization
GAE	Gallic acid equivalents
ID	Internal diameter
MS	Mass Spectrometry
NIH	National Institutes of Health
ORAC	Oxygen radical absorbance capacity
PBS	Phosphate-buffered saline
PDA	Photodiode array detector
TE	Trolox equivalents
UPLC	Ultra-Performance Liquid Chromatography
UV	Ultraviolet
