# Supplementary material for: Metabolomic Profile, Antioxidant Capacity, and Preliminary Cellular Activity of Krugiodendron ferreum (Vahl) Urb., a Traditional Plant from Yucatan
Source: Molecules. 2026 Jul 15;31(14):2478. doi: 10.3390/molecules31142478 (PMC13414239; doi:10.3390/molecules31142478)

Supplementary Figure S2. Tentatively annotated phenolic compounds

Tentative identification **Cinchonain II**

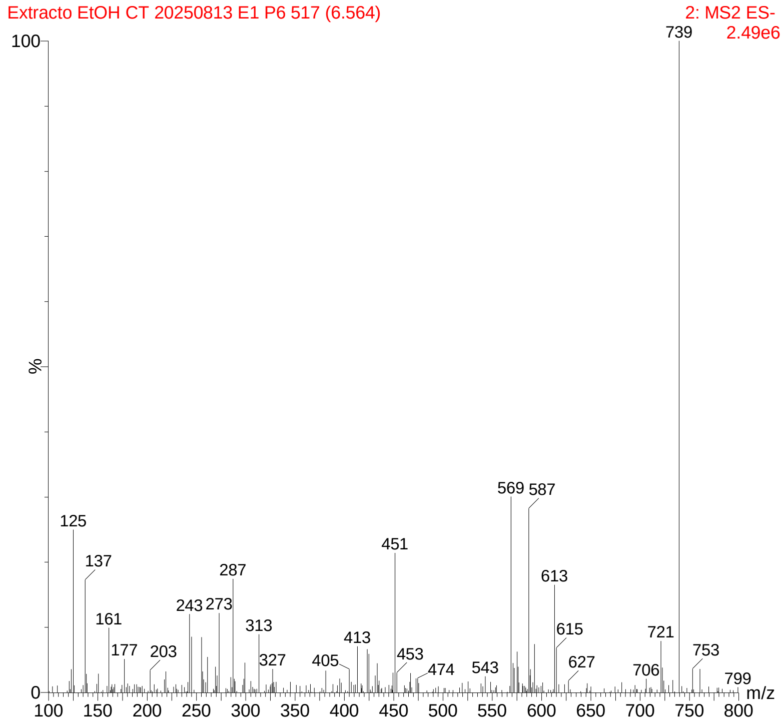

Fragmentation: 739, 723, 721, 613, 587, 569, 451, 413, 313, 299, 287, 273, 255, 245, 243, 177, 161, 137, 125

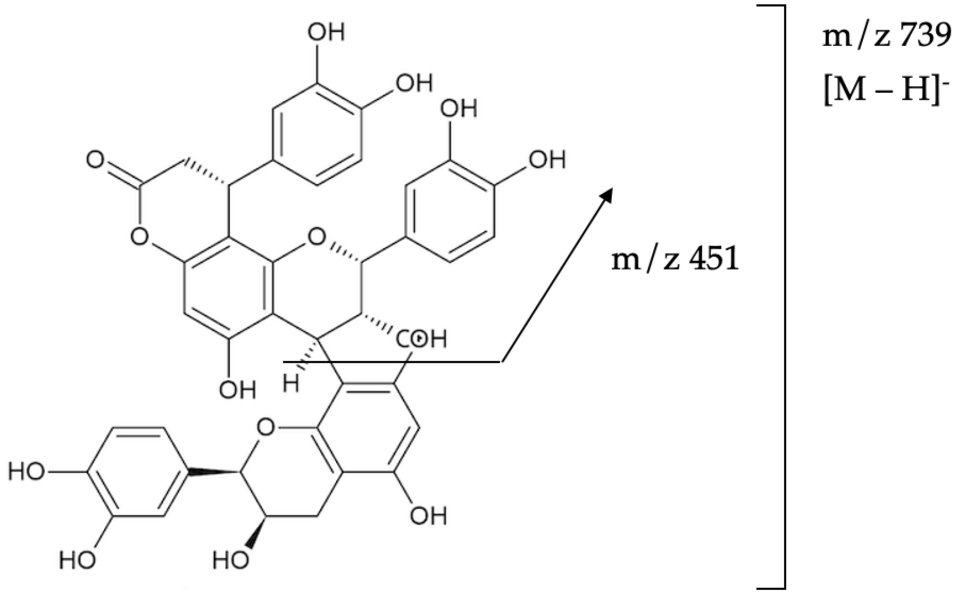

Tentative identification: **Cinchonain I**

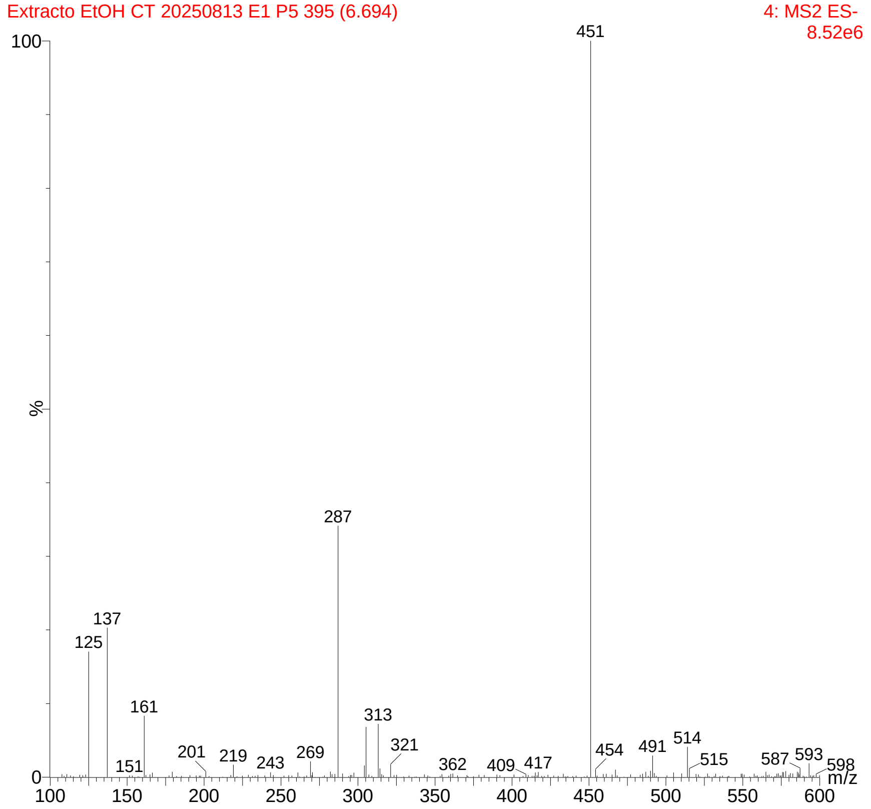

Fragmentation: 451, 313, 305, 287, 161, 137, 125

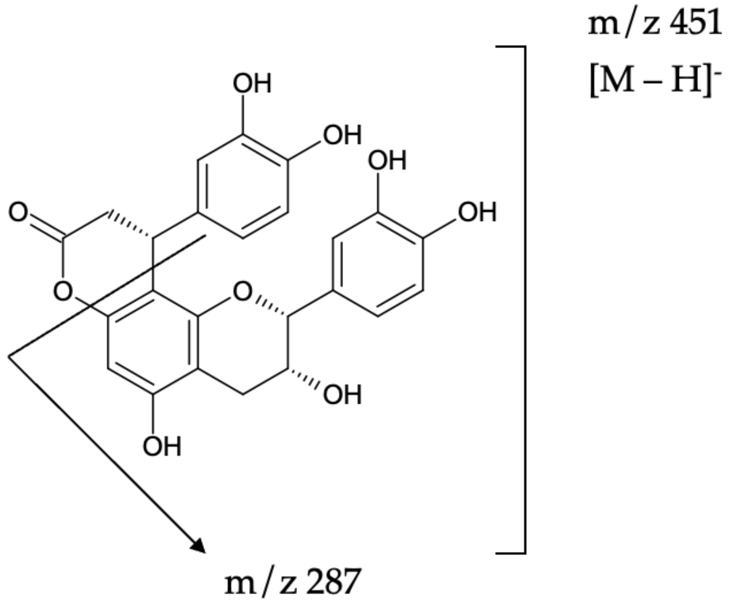

Tentative identification: **Catechin 3-O-rhamnoside**

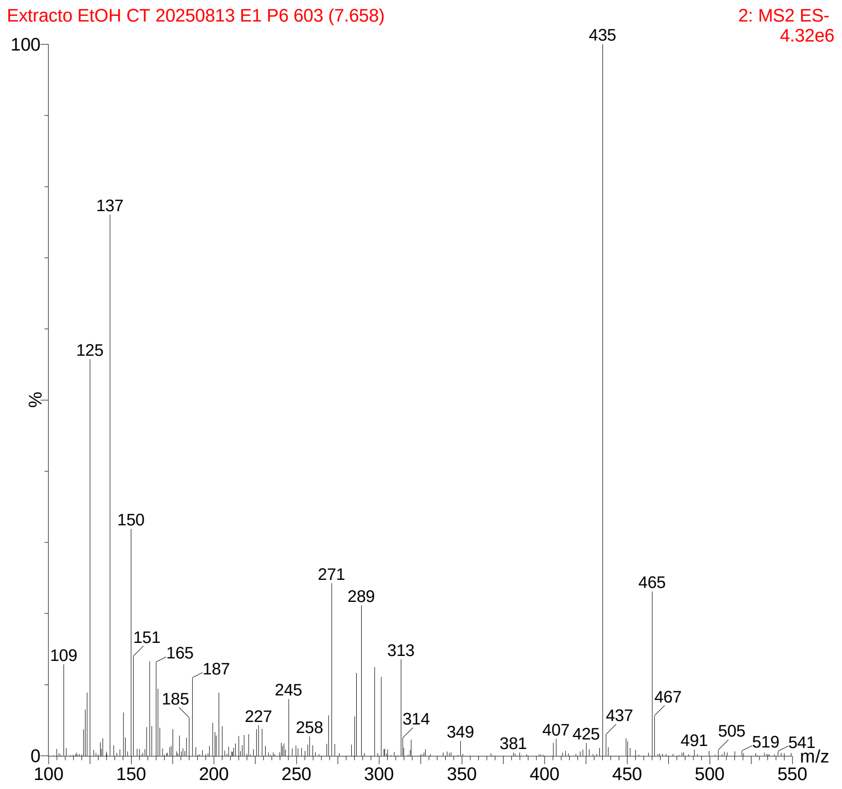

Fragmentation: 871, 435, 313, 289, 271, 245, 151, 150, 137, 125

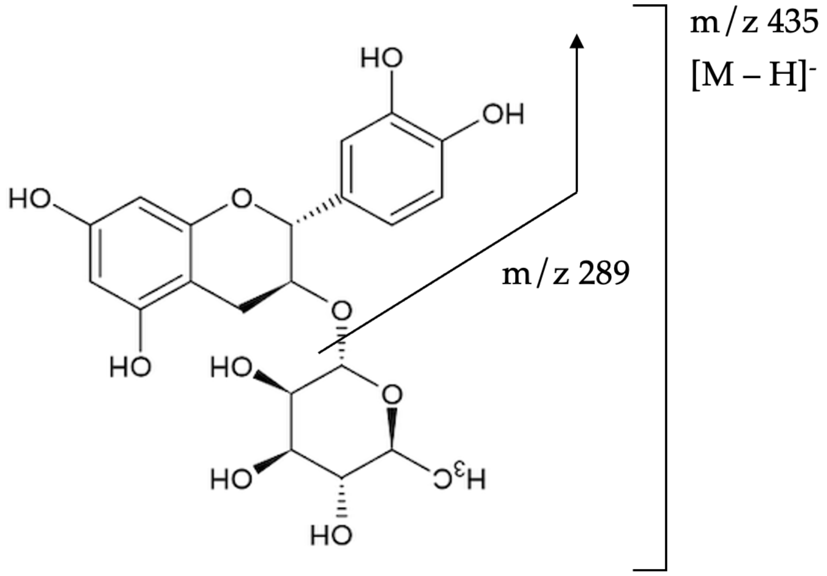

Tentative identification: **Syringoylquinic acid**

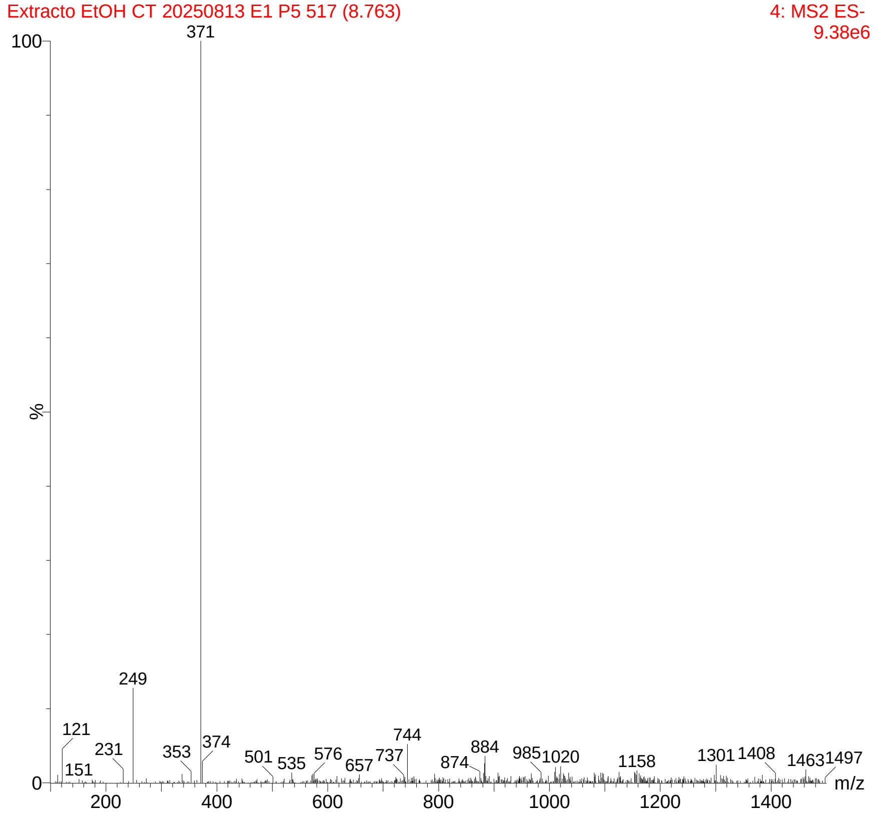

Fragmentation: 371, 249, 231

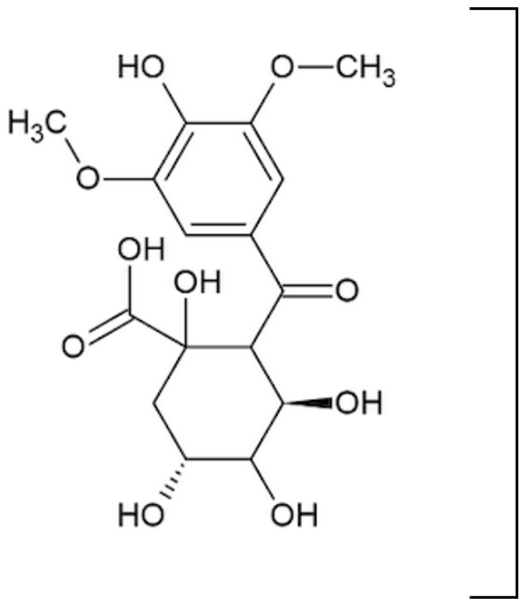

m/z 371

[M – H]-

Tentative identification: **Taxifolin 3-O-rhamnoside**

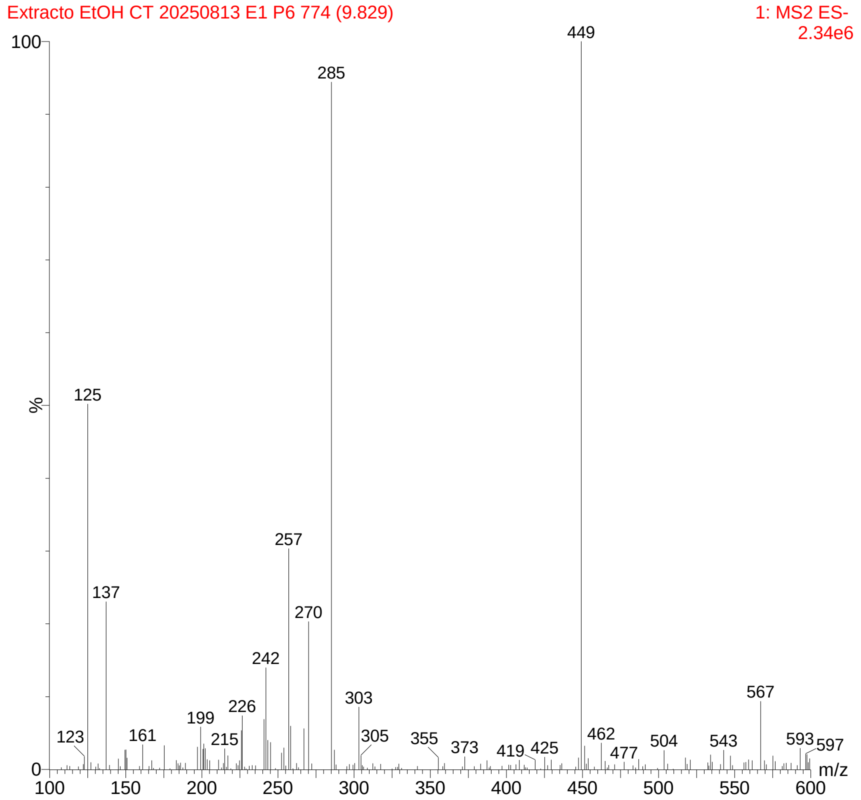

Fragmentation: 449, 303, 270, 257, 242, 226, 199, 161, 137, 125

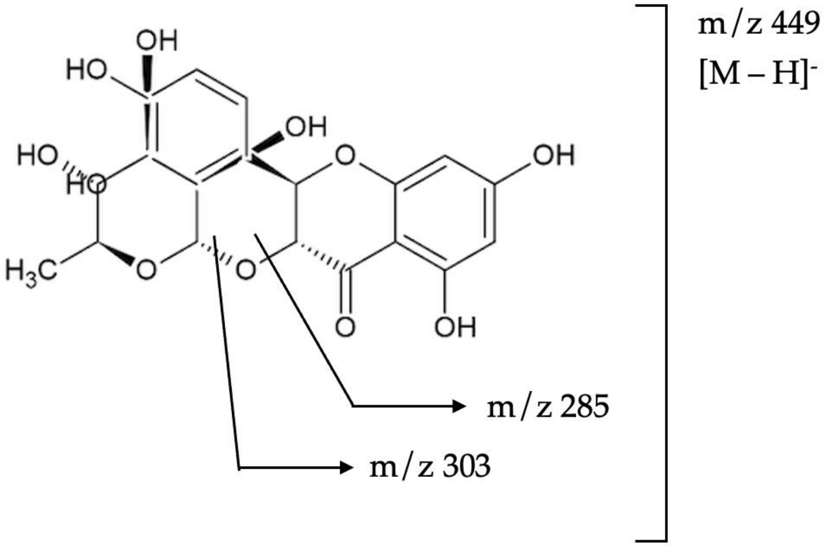

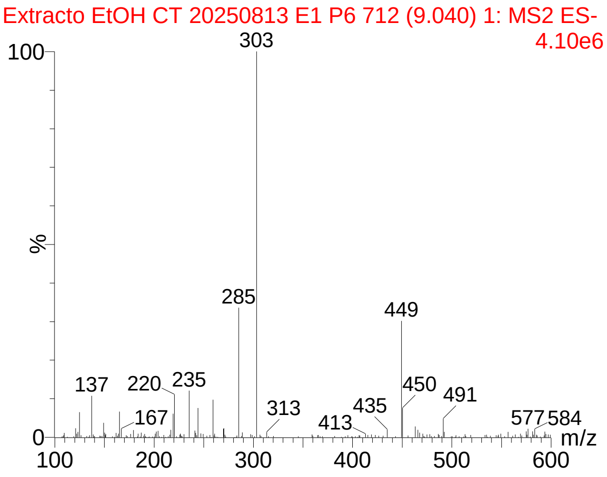

Fragmentation: 449, 285, 259, 235, 220, 219, 165, 149, 137, 125

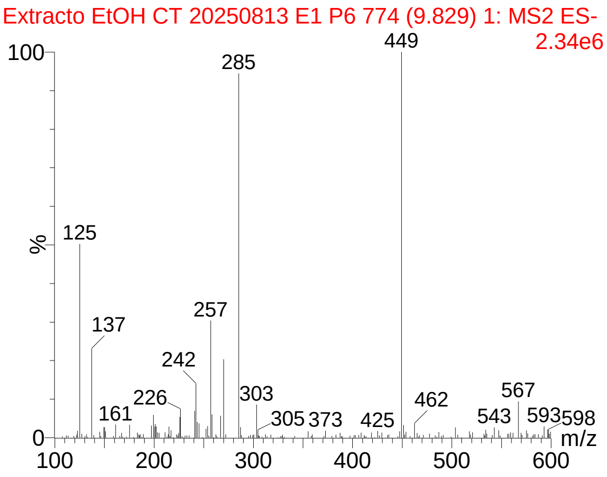

Fragmentation: 449, 303, 270, 257, 242, 226, 199, 161, 137, 125

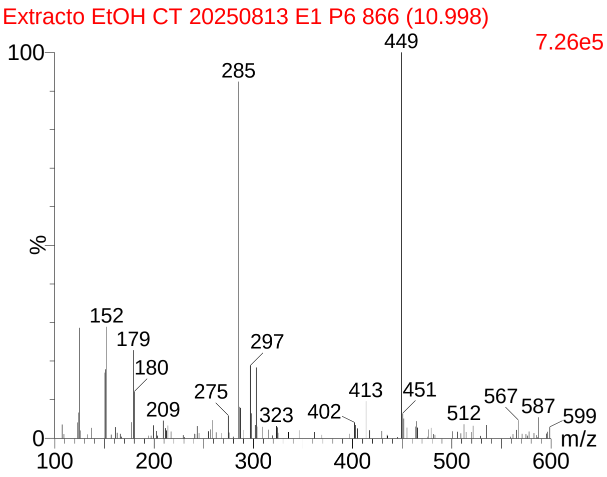

Fragmentation: 449, 303, 297, 179, 152, 151, 125

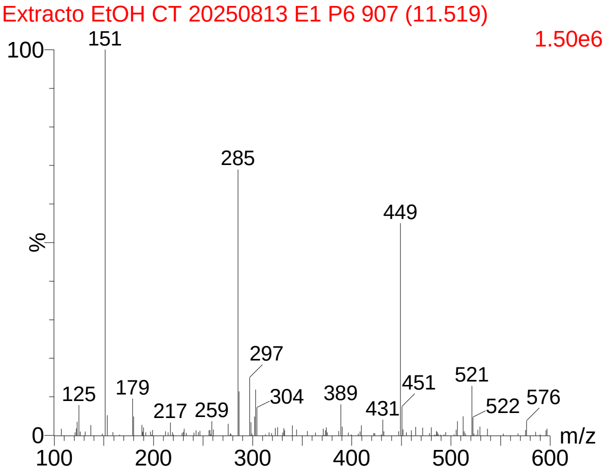

Fragmentation: 449, 303, 285, 179, 151, 125

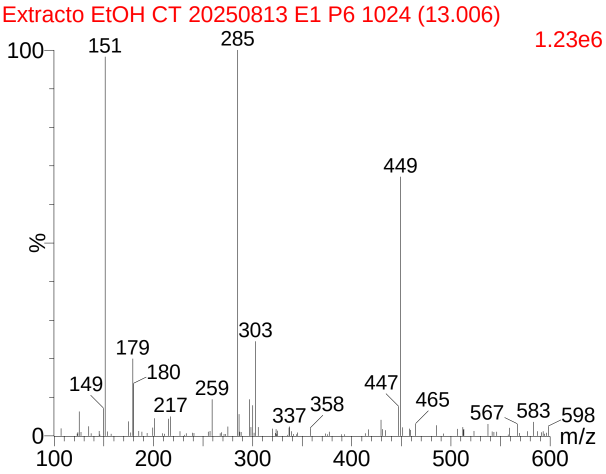

Fragmentation: 449, 303, 297, 259, 179, 125

Tentative identification: **Myricetin 3-O-rhamnoside**

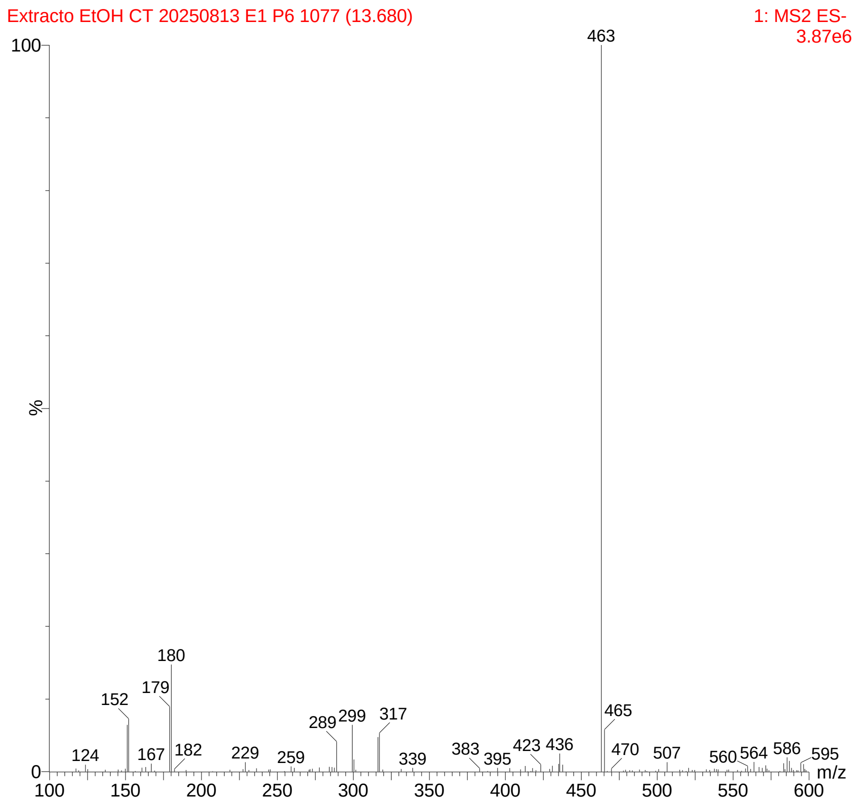

Fragmentation : 463, 299, 289, 180, 179, 152, 151

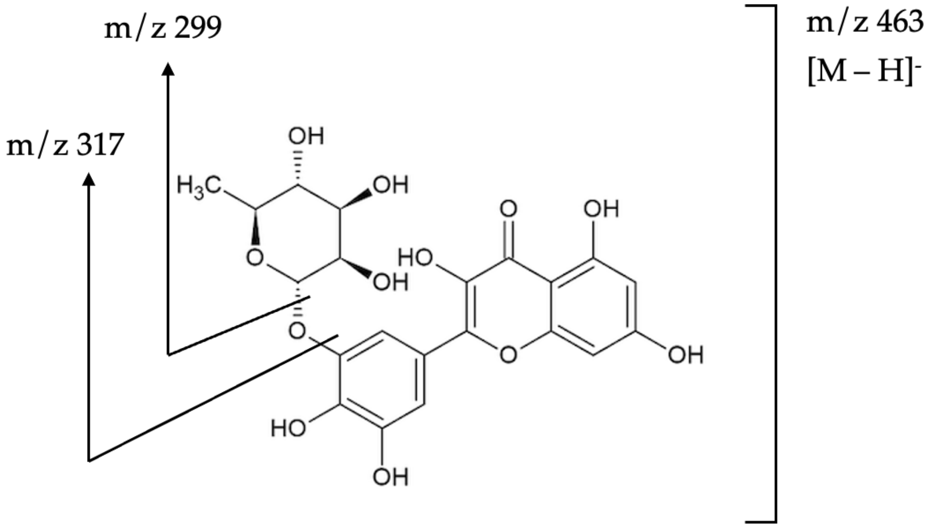

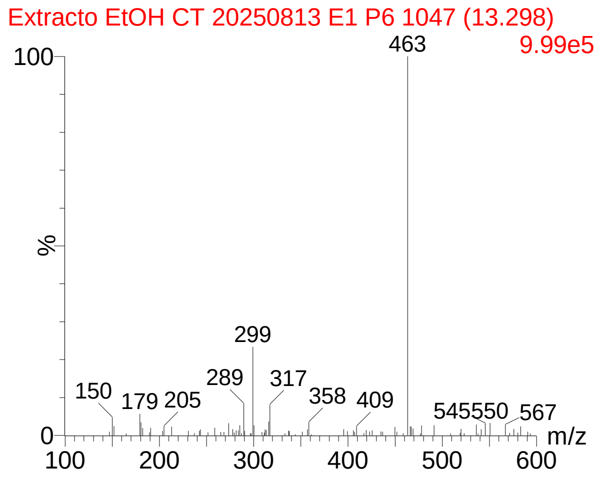

Fragmentation: 463, 317, 299, 180, 179, 152, 125

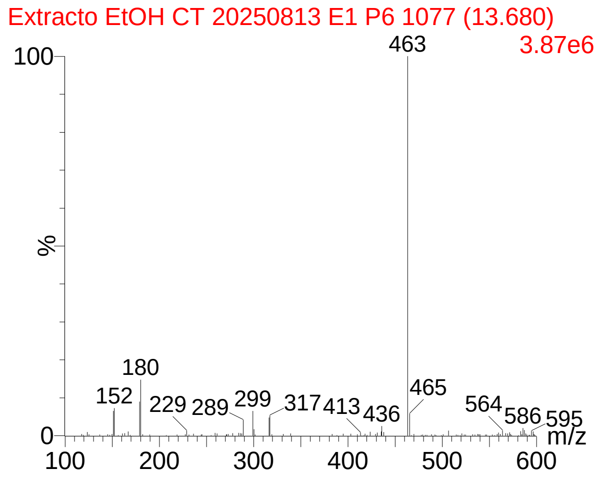

Fragmentation: 463, 317, 299, 289, 180, 179, 152, 151

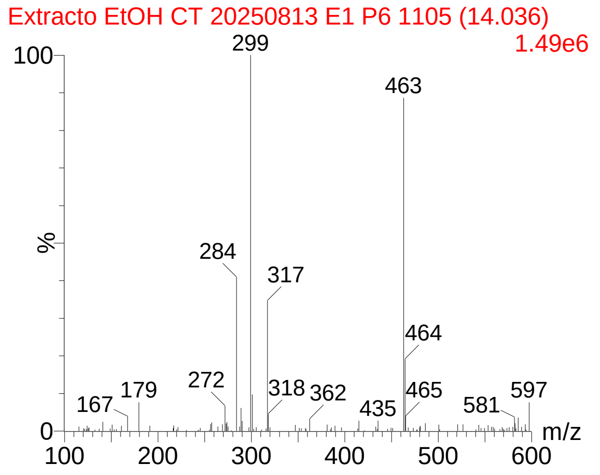

Fragmentation: 463, 317, 299, 284, 272, 179

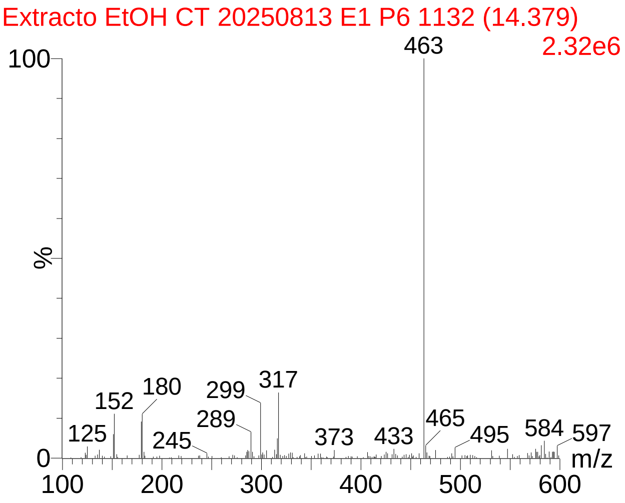

Fragmentation: 463, 317, 299, 289, 180, 179, 152, 151

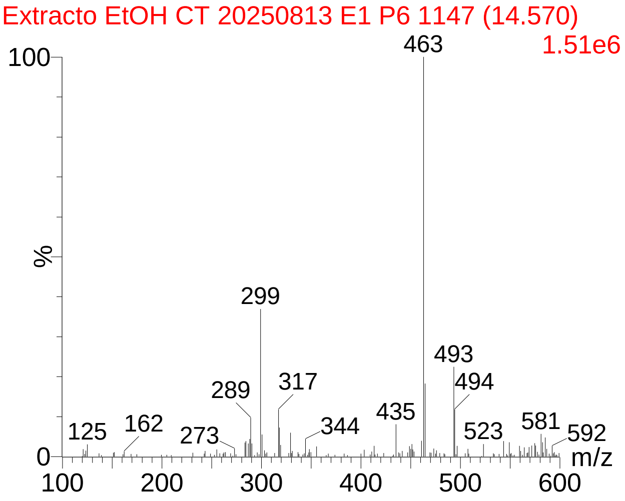

Fragmentation: 463, 317, 299, 289

Tentative identification: **Isolariciresinol 9-O-arabinoside**

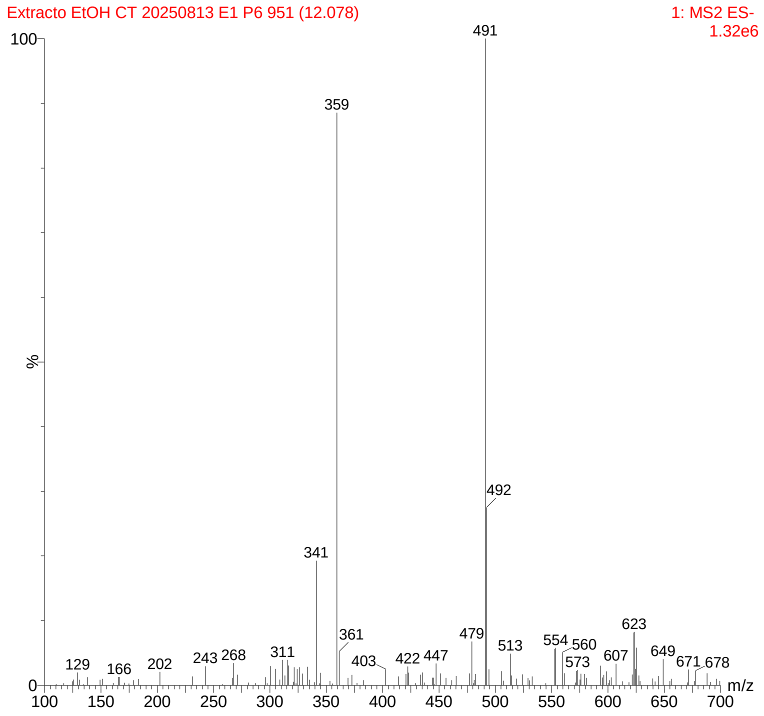

Fragmentation:491, 359, 341, 327

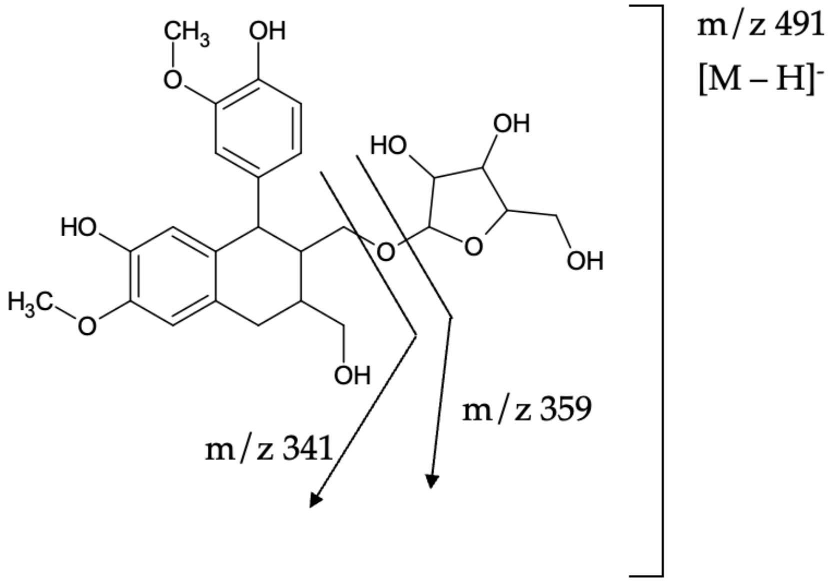

Supplement: Supplementary file 1 [file molecules-31-02478-s001.zip › Fig S2_Tentative identification.pdf]
