# Supplementary figures and images for: Metabolomic Profile, Antioxidant Capacity, and Preliminary Cellular Activity of Krugiodendron ferreum (Vahl) Urb., a Traditional Plant from Yucatan
Source: Molecules. 2026 Jul 15;31(14):2478. doi: 10.3390/molecules31142478 (PMC13414239; doi:10.3390/molecules31142478)

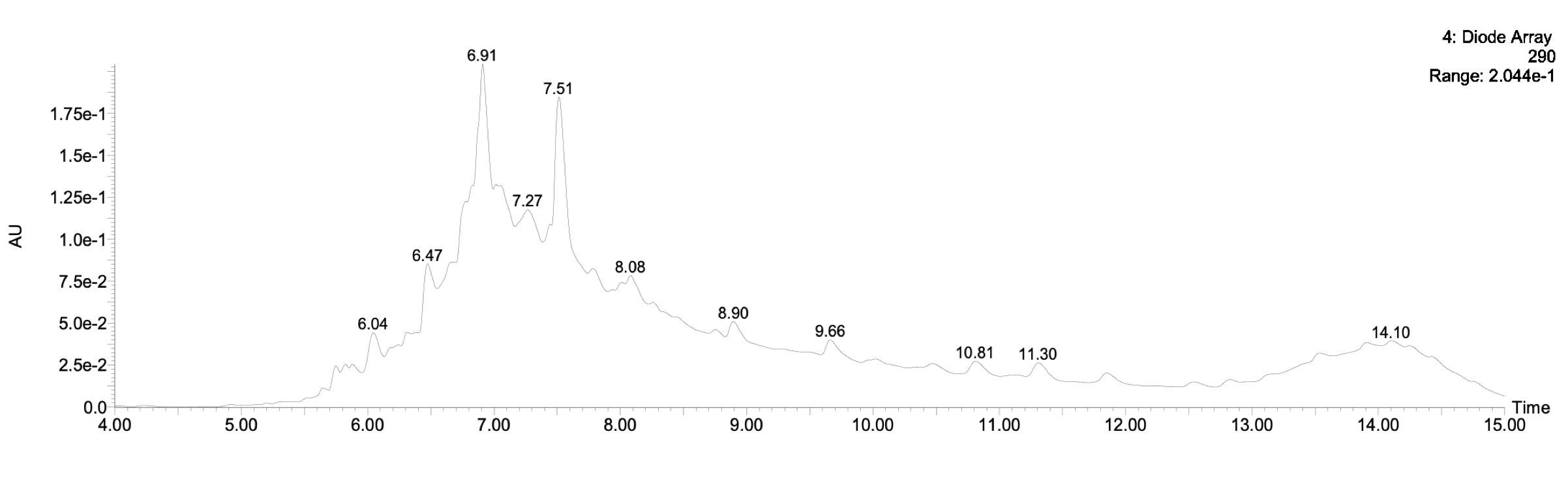

Supplement: Supplementary file 1 [file molecules-31-02478-s001.zip › Fig S1_PDA.tiff]
